# Distinguishing mechanisms of gamma frequency oscillations in human current source signals using a computational model of a laminar neocortical network

**DOI:** 10.3389/fnhum.2013.00869

**Published:** 2013-12-18

**Authors:** Shane Lee, Stephanie R. Jones

**Affiliations:** Department of Neuroscience, Brown UniversityProvidence, RI, USA

**Keywords:** gamma oscillations, pyramidal interneuronal gamma, laminar neocortex, computational neuroscience, magnetoencephalography (MEG), electroencephalography (EEG)

## Abstract

Gamma frequency rhythms have been implicated in numerous studies for their role in healthy and abnormal brain function. The frequency band has been described to encompass as broad a range as 30–150 Hz. Crucial to understanding the role of gamma in brain function is an identification of the underlying neural mechanisms, which is particularly difficult in the absence of invasive recordings in macroscopic human signals such as those from magnetoencephalography (MEG) and electroencephalography (EEG). Here, we studied features of current dipole (CD) signals from two distinct mechanisms of gamma generation, using a computational model of a laminar cortical circuit designed specifically to simulate CDs in a biophysically principled manner (Jones et al., 2007, 2009). We simulated spiking pyramidal interneuronal gamma (PING) whose period is regulated by the decay time constant of GABA_A_-mediated synaptic inhibition and also subthreshold gamma driven by gamma-periodic exogenous excitatory synaptic drive. Our model predicts distinguishable CD features created by spiking PING compared to subthreshold driven gamma that can help to disambiguate mechanisms of gamma oscillations in human signals. We found that gamma rhythms in neocortical layer 5 can obscure a simultaneous, independent gamma in layer 2/3. Further, we arrived at a novel interpretation of the origin of high gamma frequency rhythms (100–150 Hz), showing that they emerged from a specific temporal feature of CDs associated with single cycles of PING activity and did not reflect a separate rhythmic process. Last we show that the emergence of observable subthreshold gamma required highly coherent exogenous drive. Our results are the first to demonstrate features of gamma oscillations in human current source signals that distinguish cellular and circuit level mechanisms of these rhythms and may help to guide understanding of their functional role.

## 1. Introduction

Gamma frequency rhythms, described broadly anywhere from 30 to 150 Hz, are commonly observed in brain signals across many scales, regions, and species and have a well-established association with healthy function. They have been implicated in learning and memory (Osipova et al., 2006; Headley and Weinberger, 2011, 2013), attention (Fell et al., 2003; Deco and Thiele, 2009; Todorovic et al., 2011), perception (Varela et al., 1999), and plasticity (Lee et al., 2009; Headley and Weinberger, 2011, 2013) and also have been shown to be disrupted in many neuropathologies, including schizophrenia and ADHD (Haig et al., 2000; Wang, 2010). Despite the abundance of correlative evidence that gamma periodic rhythms are associated with brain function, their precise role in mediating information processing is debated (Ray and Maunsell, 2010). Furthermore, gamma periodic rhythms are most often defined by their frequency band and not by their underlying mechanism (Siegel and König, 2003), which is complicated by observations that broadband gamma activity can be, in some cases, related to secondary phenomenon such as microsaccades and not functional neuronal processes (Yuval-Greenberg et al., 2008). Critical to resolving these debates is an understanding of how specific mechanisms give rise to activity within these frequency bands (Buzsáki and Wang, 2012).

Some studies have categorized the gamma frequency band into low (30–100 Hz) and high (100–150 Hz) sub-bands (Herculano-Houzel et al., 1999; Uhlhaas et al., 2011), and further functional delineations may exist (Ainsworth et al., 2011; Uhlhaas et al., 2011). Work on understanding circuit-level origins of gamma frequency oscillations has focused primarily on the low band, and it has been well-established through experiments and computational modeling that these rhythms can emerge in local spiking networks through interactions of principal excitatory cell (E) and GABA_A_-ergic inhibitory cell (I) interactions, with the period of the oscillation set by the time constant of decay of GABA_A_-mediated currents (Cardin et al., 2009; Vierling-Claassen et al., 2010; Buzsáki and Wang, 2012), a mechanism that has been referred to as pyramidal-interneuronal gamma (PING) (Whittington et al., 2000, 2011). In normal regimes, the decay time constant of GABA_A_-mediated synapses bounds oscillations to be in the low gamma frequency band (Chow et al., 1998; Uhlhaas et al., 2011). The intrinsic biophysics of these currents suggest that they are not a candidate mechanism to underlie gamma frequency oscillations in a much higher band (Chow et al., 1998). It has been shown that increases in the high gamma band can arise from uncorrelated changes in rate of spiking (Ray et al., 2008), but whether alternative mechanisms can explain the mechanistic origin of this band is less well-understood (Ray et al., 2008; Buzsáki and Wang, 2012). One alternative hypothesis suggests that gamma frequency activity can be synaptically entrained in local cortical networks by gamma periodic inputs from an upstream region such as thalamus (Canu et al., 1994; Steriade et al., 1996b; Castelo-Branco et al., 1998).

A major confound in the interpretation of the mechanisms of gamma frequency rhythms is the scale over which they are recorded (i.e. spikes, local field potentials (LFP), electrocorticography (ECoG), electroencephalography (EEG)/magnetoencephalography (MEG)). While gamma rhythms are often thought to involve local spiking interactions that are not synchronized across large volumes of the brain (von Stein and Sarnthein, 2000; Buzsáki and Wang, 2012), macroscopic human imaging signals are separately thought to represent subthreshold synchronous activity of large populations of pyramidal neurons (Hämäläinen et al., 1993; Zhu et al., 2009). Yet gamma periodic activity is commonly reported in these studies, which raises the question of whether the underlying mechanisms are the same. This discrepancy might be partially—but not fully—rectified by the fact that the spectral power of recorded gamma is typically smaller than that of lower frequency rhythms, suggesting gamma rhythms are generated by a smaller synchronous network. Yet how these large scale observations in human imaging can be reconciled with the spatially restricted operation of some modes of gamma oscillations remains unclear.

In prior work, numerous computational neural models have been created to study the mechanisms of only local gamma frequency rhythms in spiking networks, focusing on E-I (or I-I) interactions that generate low gamma via GABA_A_-mediated mechanisms (Traub et al., 1996, 1997; Chow et al., 1998; Börgers et al., 2005), and to our knowledge, these studies have not considered gamma generating mechanisms or alternatives explicitly in the context of current dipole (CD) signals commonly estimated with MEG/EEG. If designed at the appropriate scale to accurately simulate the physics of the recorded signals, computational models can elucidate features of the rhythms that help to disambiguate the interpretation of the underlying mechanisms.

In this paper, we simulated different mechanisms of gamma frequency rhythms in CD using a previously developed, biophysically principled computational model of a laminar neocortical circuit designed specifically to simulate macroscopic CD signals estimated from MEG or EEG recordings (Jones et al., 2007, 2009; Ziegler et al., 2010). Our model results predict features in CD waveforms that would arise from gamma frequency rhythms created by GABA_A_-mediated spiking networks and by subthreshold driven networks. We show that there are quantifiable features in the raw waveforms that can help to differentiate the underlying mechanisms. High gamma frequency activity emerged from a characteristic CD waveform produced by spiking network rhythms that was non-harmonically coupled to low gamma frequency rhythms and was not representative of a separate process or uncorrelated spiking activity. Gamma frequency rhythms in CD were dominated by the long apical dendrites of pyramidal cells in infragranular layer 5 (L5) that can mask the presence of independent gamma rhythms in supragranular layers 2/3 (L2/3). Spiking-mediated gamma and subthreshold driven gamma were coordinated by networks of different scales, with smaller spiking networks creating higher amplitude signals. Our findings provide the first principled interpretation of the cellular-level mechanisms of gamma in human macroscopic imaging signals that may help reveal the computational role of gamma in health and disease.

## 2. Methods

We utilized a prior biophysically-based computational model a laminar cortical circuit designed to simulate current dipoles (CD) (Jones et al., 2007, 2009; Ziegler et al., 2010). Here, we briefly describe key features of the model and differences in the present work, which were necessary to study gamma rhythms specifically.

### 2.1. Model network construction

**Figure 1:**
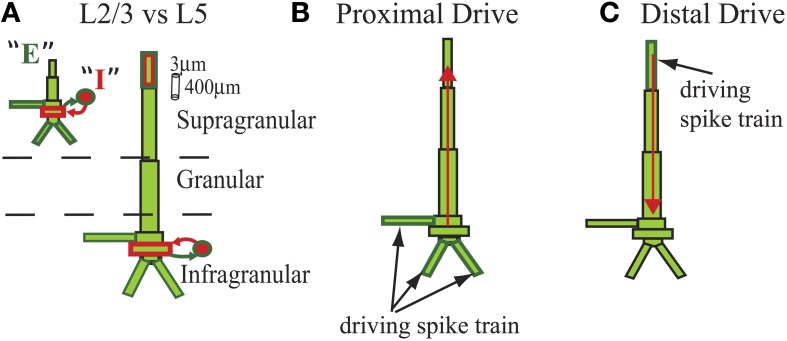
**Schematic of cells in model laminar cortical network.** Multi-compartment excitatory pyramidal cells (E, green) and single compartment inhibitory basket cells (I, red) shown for cells with somata in supragranular L2/3 and infragranular L5 (E and I labeled for L2/3 cell but represented similarly for L5). Rectangles denote compartments. Cylindrical scale bar marks 3 μm diameter and 400 μm length. Figure adapted from (Jones et al., 2007, 2009). **(A)** Lines between cell types denote GABA_A_-ergic synaptic connections from I cells (red) and AMPA-ergic synaptic connections from E cells (green) that terminated on somatic compartments. **(B)** In some simulations, all L5 pyramidal cells received identical subthreshold proximal input, modeled as AMPA-ergic excitatory postsynaptic currents (EPSCs) that were delivered simultaneously to three compartments in each cell, as shown. **(C)** In some simulations, all L5 pyramidal cells received identical subthreshold distal input, also AMPA-ergic, and delivered to distal compartment of apical dendrites. Red arrows illustrate examples of longitudinal intracellular current dipole induced by each drive.

The model represented the laminar structure of a neocortical column simulated with two major cell classes in supragranular layer 2/3 (L2/3) and infragranular layer 5 (L5). Each layer was comprised of 100 multi-compartment pyramidal cells and 35 fast spiking single compartment inhibitory cells (Figure 1) that were arranged in a grid.

### 2.2. Model cell morphology and physiology

Pyramidal cell morphology is particularly relevant to the present work. As previously described, the compartmentalization of the pyramidal cells was modeled to mimic a reduced morphology in neocortical pyramidal neurons (Bush and Sejnowski, 1993; Jones et al., 2009). The compartment lengths and diameters are as reported in Jones et al. (2007). In general, all pyramidal cells in both layers contained a somatic compartment, apical dendritic compartments that were oriented orthogonally to the laminae, an oblique dendritic compartment parallel with the laminae, and 3 basilar dendritic components, as depicted in Figure 1. The oblique dendritic compartment did not contribute to the CD calculation in the model, but all others did. L5 pyramidal cells included one additional apical dendritic compartment and were in total 2.21 times longer than the L2/3 compartments for equivalent CD. The L5 and L2/3 pyramidal compartments had differences in compartmental diameter and capacitance, which were identical to prior work and can be found there.

For some compartments with lengths greater than 100 μm (L2 pyramidal—oblique, middle apical dendritic section, apical dendritic tuft; L5 pyramidal—apical dendritic trunk, apical tuft), the spatial discretization used for integration within a compartment was increased by 1 additional point. For these models, this minor modification had negligible numerical effects and did not affect the results of the simulations.

All cells in the model contained Hodgkin-Huxley type sodium (I_Na_), potassium (I_K_), and leak (I_L_) currents. Additional active currents in pyramidal cells in L2/3 included a delayed rectifier potassium current (I_Kdr_), and a potassium M current (I_Km_). Pyramidal cells in L5 also contained these currents, in addition to a calcium current (I_Ca_), mixed cation h current (I_h_), a low threshold T-type calcium current (I_CaT_), and a potassium-activated calcium current (I_KCa_). All parameters for these currents, including maximal conductances and reversal potentials, were identical to Jones et al. (2009).

### 2.3. Synaptic connectivity

The types of connections from the original model (Jones et al., 2009) were preserved and included AMPA-ergic and GABA_A_-ergic synapses, but the intralaminar synaptic connectivity strengths between cell types were modified for the present work. We briefly describe the details of the intralaminar GABA_A_-ergic synapses due to their importance in mediating spiking gamma frequency rhythms. GABA_A_-ergic synapses terminated on the somatic compartments of pyramidal cells, which were modeled with an exponential rise time constant of 0.5 ms and an exponential decay time constant of 5 ms, as in Jones et al. (2009). Synaptic connections between cell types were effectively all-to-all, but the strengths were scaled by a weight space constant that considered the distance between cells in the grid.

A common view of low frequency gamma oscillations is that they emerge from synchronous postsynaptic currents in populations of pyramidal neurons, including both GABA_A_-ergic and glutamatergic synapses. Our model is consistent with this view while explicitly considering effects of these synaptic interactions on intracellular longitudinal currents in pyramidal neurons that contribute to current source signals. However, we have not included E-E connections in our study. This simplification was chosen based on prior studies showing the minimal synaptic connectivity to achieve PING rhythms includes reciprocal interactions between E and I cell populations (Whittington et al., 2000; Börgers et al., 2005). Though E-E connections play an important roles in network dynamics, here we were interested in the effect of GABA_A_-ergic mediated PING rhythms (via I-E) on the current dipole signal. Prior studies have shown that the addition of E-E connections can maintain PING rhythms provided the I-E connections are sufficiently strong (Börgers and Kopell, 2003; Tiesinga and Sejnowski, 2009), and we expect a consistent parameter regime could be achieved in our model, though future studies would be needed to explicitly address this question. Interlaminar connections between cell types in different laminae were also removed for these simulations, in contrast to prior models, because our focus here was on independent oscillatory activity in L2/3 and L5 (see Discussion).

### 2.4. Current dipole calculation

The longitudinal CD was the principal output of the simulations. The aggregate CD was calculated as the linear sum of the longitudinal intracellular dendritic currents from L2/3 and L5 pyramidal cells in a direction parallel to the apical dendrites. This signal is schematically depicted with red arrows in Figures 1B,C. In most cases, CD was analyzed separately for L2/3 and L5.

The CD for a given layer (Q_total_) was the sum over all pyramidal cells in a layer (n_*pyr*_) of the axial CD contribution for each compartmentalized segment *i*, which was the ohmic current between adjacent segments multiplied by the length component of the segment parallel to the longitudinal axis of the apical dendrite (*z*_*i*_). Thus, Q_total_ at each time point for a given layer was calculated as:

$$Q_{\text{total}}=\sum_{n_{pyr}}\sum_{i,j}\left(\frac{v_{i}-v_{j}}{r_{i}}\right)\cdot z_{i}$$

for which *r*_*i*_ was the axial resistance and *v*_*i*_ was the voltage of the *i*th segment, and *v*_*j*_ was the voltage of the preceding segment *j*. The *z* axis was always defined parallel to the apical dendrites, and the positive direction was defined in the direction corresponding to a net current flow traveling away from the soma toward the local cortical surface.

### 2.5. Parameters for simulations of PING and weak PING

In this paper, we simulated an established class of spiking mechanisms of gamma rhythms that rely on the interactions between pyramidal cells and interneuronal cells, so-called PING rhythms (Whittington et al., 2000). In general, PING rhythms are initiated by excitation to the E cells, which in turn synaptically activates a spiking population of I cells. In turn, these I cells inhibit the E cells, shunting further E cell activity until the excitatory drive to the E cells can overcome the effects of the inhibition. The frequency of the rhythm is paced by this time constant of inhibition (Whittington et al., 2000; Cardin et al., 2009; Whittington et al., 2011; Buzsáki and Wang, 2012), which is mediated by GABA_A_-ergic currents and is typically studied in two forms: strong and weak. Principal E cells participate on each cycle of the strong PING rhythm, while only a sparse, random set participate on each cycle in weak PING. Computationally, strong and weak PING rhythms differ in the type and strength of excitation and the maximal synaptic strengths of connections between excitatory principal cells and inhibitory interneurons. We considered strong PING rhythms briefly in this paper but focused on weak PING in CD.

Strong PING rhythms were created by tonic applied current (I_app_) to the E cells, which was manifested as the addition of a scalar current term in the dynamic current balance equations for the compartmental voltage. The maximal conductance of AMPA-ergic synapses from E to I cells (g_*ei*_) was tuned such that a coherent barrage of E cells was required from the whole population to elicit coherent spiking in a group of I cells. The maximal conductance of GABA_A_-ergic synapses from I to E cells (g_*ie*_) was tuned such that the coherent population of I cell spiking would shunt further E cell activity. No I to I connections were used for strong PING simulations.

Weak PING rhythms were modeled as noisy, Poisson-distributed AMPA-ergic excitatory postsynaptic potentials (EPSPs) with rate parameter (λ_*pois*_) and maximal synaptic conductance (g_*pois*_) to each individual E cell, as in Börgers et al. (2005). The network was tuned such that a random population of E cells elicited spiking in a population of coordinated I cells by increasing g_*ei*_. For inhibition, g_*ie*_ was tuned to shunt further random E cell spiking. In weak PING, I to I synapses were used to increase coherence in the I cell spiking, tuned by a maximal conductance (g_*ii*_). The parameters used in simulations of both strong and weak PING are shown below in Table 1 for different layers, unless otherwise noted in the text. Units for conductances are in mS/cm^2^, and units for currents are μA.

**Table 1:** **Parameters for PING**.

	**g_ei_**	**g_ie_**	**g_ii_**	**I_app_**	**λ_Pois_ (Hz)**	**g_pois_**
L5: strong PING	6×10^−5^	3×10^−1^	0	6	–	–
L5: weak PING	9.1×10^−4^	8×10^−2^	7.5×10^−3^	–	40	1×10^−2^
L2: weak PING	1.2×10^−3^	7×10^−3^	1×10^−2^	–	140	0.8×10^−3^

For all oscillations simulated in the network, specific frequencies were chosen to fall within the canonical gamma frequency band, supported by experimental evidence of gamma during working memory (Howard, 2003), evoked by sensory stimuli (Tavabi et al., 2011; Swettenham et al., 2013), and induced by attention (Koelewijn et al., 2013). In our simulations, weak PING rhythms were frequency-matched to the subthreshold oscillations described below. For all parameter regimes, oscillatory behavior was modulated by the strength of the excitation, and changing the balance of parameters could affect the observed network frequency, as in previous studies (Ainsworth et al., 2011).

### 2.6. Subthreshold driven oscillations

We simulated subthreshold oscillations driven from sources outside of the cortical network, representative of thalamocortical or corticocortical synaptic drives to cortex, as in our prior work (Jones et al., 2009). Inputs were simulated as predefined spike trains that drove excitatory AMPA-ergic currents in the model, in distinct laminar locations shown in Figures 1B,C and referred to as “proximal” and “distal.” Proximal inputs reflected lemniscal thalamocortical excitation (but see Discussion) that contacted granular and infragranular layers and ramified with synaptic locations in three compartments on the L5 pyramidal cells: the apical oblique dendrite and both of the basal dendrites attached to the basal trunk. Distal inputs were modeled to reflect corticocortical activity or thalamic sources and ramified at the most superficial distal dendritic compartment on the pyramidal cells.

Each type of input was modeled to be the same for all cells in a given simulation. Inputs were parameterized by mean inter-event intervals that were chosen to be within gamma frequency ranges for these simulations. The event times served as the mean time for 10 Gaussian distributed excitatory postsynaptic currents (EPSCs) with a temporal standard deviation (σ_*p*_ for proximal inputs and σ_*d*_ for distal inputs) that was varied. A delay in the mean time between proximal and distal inputs for each cycle was set to 5 ms for all simulations presented here. In previous modeling, a synaptic delay was introduced between proximal and distal inputs to reflect a delay in thalamocortical transmission, but here both delays were set equal to 1 ms for all subthreshold simulations for simplicity. The conductances of all subthreshold inputs were set to 40 pS, as used for certain simulations in prior work.

### 2.7. Analysis

Spike times were recorded for all cell types, as well as somatic current contributions from GABA_A_-mediated synapses onto pyramidal cell somata using NEURON's built-in routines. Spectrograms were calculated using a Morlet wavelet method as previously described in Jones et al. (2009); Vierling-Claassen et al. (2010) over a frequency range of 1–150 Hz. Units of Morlet power (P_M_) are (nAm)^2^. We also used a stationary Welch periodogram from the SciPy signal module (Oliphant, 2007) to characterize the spectral content for the entire time window of a simulation of CD (Hann tapered). Units of Welch power spectrum (P_W_) are (nAm)^2^.

**Figure 2:**
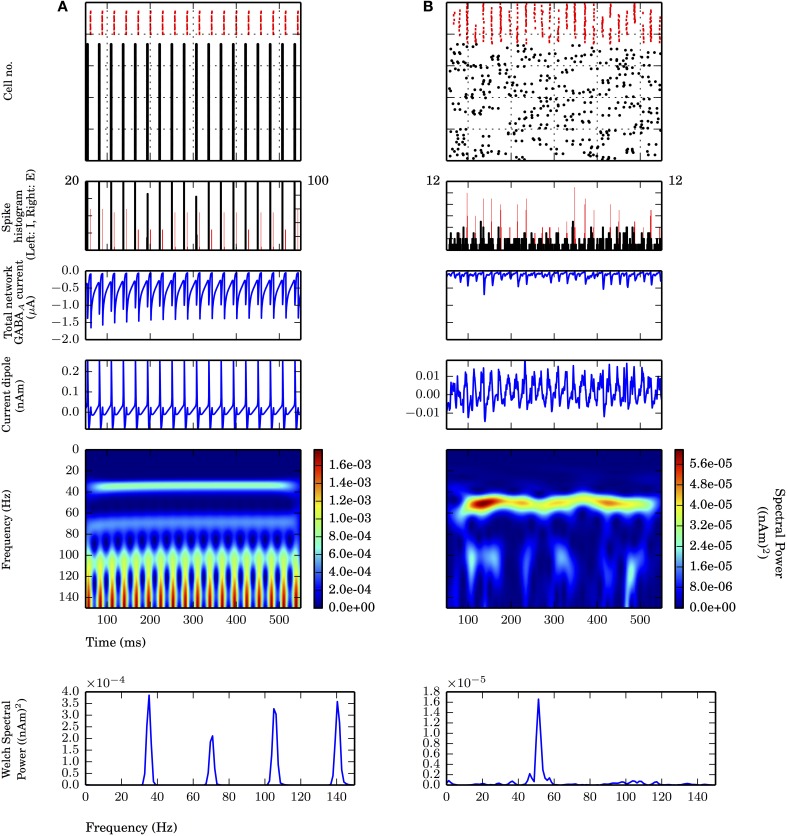
**Strong PING and weak PING rhythms have characteristically different current dipole waveforms.** Simulations from L5 network consisted of 100 E cells and 35 I cells, synaptically connected as described in Methods. From top panel to bottom: spike raster of E cells (black) and I cells (red); spike histogram of E cells (black, right scale) and I cells (red, left scale), 1 ms bins; aggregate GABA_A_-ergic current contribution to E cell somata (μA); network current dipole (nAm); Morlet spectrogram of current dipole; stationary Welch periodogram for current dipole. Simulations shown from 50 to 550 ms. **(A)** Strong PING oscillations with all pyramidal cells spiking on each cycle of the oscillation exhibit large, coordinated current dipoles. PING oscillation frequency (*f*) is 36 Hz. Individual pyramidal cells spiking at *f*. Sharp edge effects seen in broadband high frequency noise on each cycle in Morlet spectrogram. Max. amplitude of current dipole of 0.26 nAm. Max. Welch spectral power (P_W_) of 3.86×10^−4^ (nAm)^2^ at 35.4 Hz. **(B)** Weak PING oscillations have fewer E cells spiking on each cycle, despite coordinated I cell spiking activity. Fewer and less coordinated E cell spiking resulted in smaller amplitude current dipole. g_*pois*_ = 0.75×10^−2^ mS/cm^2^. Max. amplitude of current dipole of 0.019 nAm. Max. Morlet frequency at 52 Hz. Max. P_W_ of 1.66×10^−5^ (nAm)^2^ at 51.3 Hz.

Spike histograms were shown for model network cell spikes for some simulations were calculated using 1 ms bins (see Figure 2). Peristimulus time histograms (PSTH) were shown in some simulations for discrete events (such as input times) with 5 ms bins (Δt_*bin*_) unless otherwise noted.

**Figure 3:**
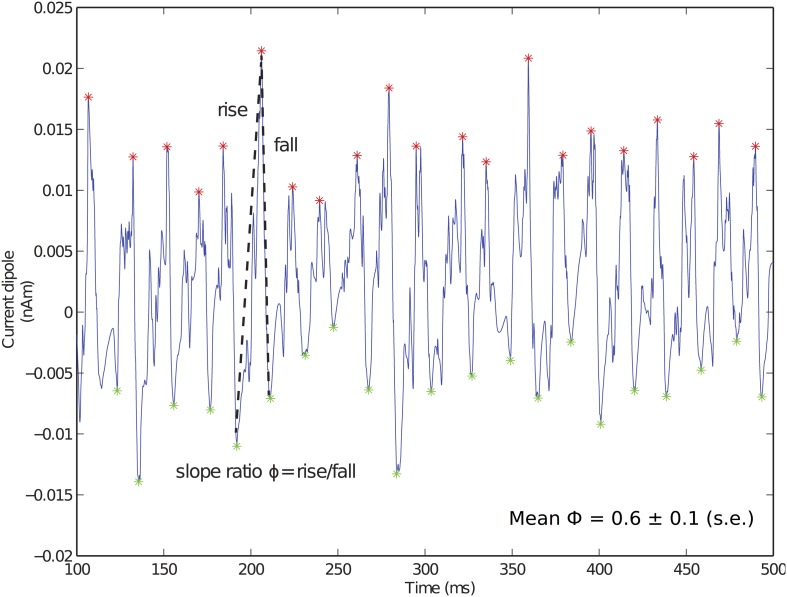
**Fast inhibition creates sharp falling phase of current dipole in weak PING.** Example weak PING current dipole (nAm) simulation, with peaks (red) and troughs (green) marked. Slope ratio (Φ) defined as mean of ratios of rising slope to falling slope (depicted by dotted lines) for each cycle of rhythm. Φ for weak PING was 0.6 ± 0.1 (s.e.), reflecting sharp inhibition responsible for rapid fall. g_*pois*_ = 0.75×10^−2^ mS/cm^2^.

Local maxima and minima of the CD were calculated for both spiking PING and subthreshold oscillations as in Jones et al. (2009) and used here to quantify slope ratio (Φ), as shown schematically for Figure 3. For the *i*th cycle of the oscillation, a ratio describing the rising rate and falling rate of the oscillations (ϕ_*i*_) was defined as ϕ_*i*_ = |*m*^*rise*^_*i*_|/|*m*^*fall*^_*i*_| in which *m*^*rise*^_*i*_ and *m*^*fall*^_*i*_ were linear slope calculations between the trough and the peak on subsequent cycles of the oscillation. Φ served as a measure of symmetry about a peak of the CD, with values of 1 suggesting that the rate of rise was equivalent to the rate of fall. Value of Φ less than 1 suggested that the rate of rise was slower than the rate of fall; conversely, values greater than 1 suggested that the rate of rise was greater than the rate of fall.

### 2.8. Numerics

Simulation data was output at a fixed time step of 0.025 ms, using the variable time step built-in NEURON solver “cvode.” All simulations shown here were run for 550 ms of simulation time, and all figures are presented for analyses run from the last 500 ms. The first 50 ms of all simulation data was ignored due to a non-linearity in the steady state initial conditions. Additionally, a baseline renormalization was performed on the CD time series for visualization purposes. For each pyramidal cell in L2, an offset of 4.43×10^−8^ nAm was applied. For each pyramidal cell in L5, over the temporal interval used in the analysis, the line 1.01×10^−10^*t* − 4.841×10^−5^ nAm was subtracted for times *t*. This linear shift did not affect the simulation results or the subsequent analyses.

The original NEURON hoc code (ModelDB accession number 136803) from Jones et al. (2009) was refactored using NEURON 7.3 as a module for Python 2.7.5. Simulations and analyses were performed in Python, using NumPy 1.7.1, SciPy 0.12.0, mpi4py 1.3, and OpenMPI 1.6.1 on commercially available x86_64 hardware. The code for these simulations is available on ModelDB (Hines et al., 2004) with accession number 151685.

## 3. Results

### 3.1. PING in current dipole signals reflects dendritic backpropagation of action potentials and strong somatic inhibition

Gamma rhythms created by well-established PING mechanisms have been observed in many models and *in vitro* and *in vivo* experiments, but how these mechanisms are expressed in macroscopic current dipole (CD) signals commonly estimated with MEG/EEG has not been investigated. To address this, we simulated PING rhythms in a laminar model of neocortex designed specifically to investigate CD signals in a biophysically based model (Jones et al., 2007). We show below that strong and weak PING rhythms created distinguishable characteristics in their CD waveforms that can help to guide the interpretation of the underlying cellular and network level generators and differentiate them from alternate mechanisms, such as driven subthreshold oscillations as described below.

First we simulated strong PING rhythms in a L5-only network. As in classical models of PING, the network model consisted of 100 excitatory pyramidal neurons (E cells) and 35 fast spiking inhibitory basket cells (I cells) coupled via AMPA and GABA_A_-mediated synaptic connections in their somatic compartments (see Figure 2A and Methods for details of parameters). Tonic, depolarizing current was simulated in the somatic compartments of the E cells to generate spiking. The typical spiking pattern in the E and I cells representative of a strong PING rhythm is shown in Figure 2A (top panel). Following prior work, we use the term “strong PING” to reflect the fact that the entire population of E cells spiked coherently on each cycle of the oscillation. This highly coherent activity is visible in both the spike rasters and histograms in Figure 2A (second panel). While such tight coherence is likely unrealistic, an investigation of the impact of strong PING rhythms was instructive in understanding the mechanisms of this rhythm in CD sources.

The rhythm was initiated by the population of E cells spiking synchronously, triggering synchronous spiking in the I cells via the AMPA-ergic E-I synapse. The spiking of the I cells led to GABA_A_-mediated synaptic activity that suppressed further E cell activity until the inhibition wore off, enabling the tonic depolarization to initiate the next cycle of E cell spikes. The time constant of the GABA_A_-mediated synapse regulated the period of the rhythm. Aggregate GABA_A_-ergic synaptic currents onto the E cell somata shown in Figure 2A verified the influence of inhibition-mediated rhythmicity.

While the underlying mechanisms of PING rely on spiking activity in E-I populations, primary CD signals arise from the intracellular longitudinal currents within the long, parallel apical dendrites of neocortical pyramidal neurons (Hämäläinen et al., 1993; Ikeda et al., 2005; Jones et al., 2007). Figure 2A (middle panels) shows the corresponding net current dipole moment in the E cell population during strong PING (see Methods for details of CD calculation).

The CD waveform was qualitatively different than that of the somatic GABA_A_-mediated synaptic currents in the E cells and the spiking activity, though they all shared similar frequency characteristics in this extreme case of strong PING. The CD consisted of rapid, large amplitude positive deflections, followed by rapid, downward negative deflections (see Methods for CD sign convention). Positive deflections reflected backpropagation of action potentials through apical dendrites of the synchronously spiking population of E cells, and negative deflections were a result of rapid current flow down the dendrites, created by the GABA_A_-ergic currents at the soma. The spectral content of this oscillation in CD included the 36 Hz gamma frequency oscillation reflective of PING mechanisms, which was corroborated by counting cycles of the oscillation in the spiking raster, somatic current, and the CD (18 cycles over 500 ms). Harmonic activity (72 Hz, 108 Hz) emerged due to the absence of cycle-to-cycle variance in this idealized strong PING rhythm. Furthermore, high frequency, broadband spectral energy dominated the spectrum on each cycle of the oscillation, corresponding to spectral edge effects caused by the sharpness reflected in the CD time series (Kramer et al., 2008; Scheffer-Teixeira et al., 2013).

Due to the fact that there is a one-to-one correspondence in units of measure for equivalent current dipole signals estimated from MEG/EEG data and our simulations, namely nanoampere-meters (nAm), we can estimate the size of the synchronous spiking network of pyramidal neurons that would create an observable CD arising from a strong PING rhythm. Estimates of minimal measurable cortical generators of CD signals in humans are on the order of 10 nAm (Hämäläinen et al., 1993; Murakami and Okada, 2006; Jones et al., 2007; Nevalainen et al., 2008; Jones et al., 2009, 2010). Here 100 E cells spiking synchronously created a CD with a maximal value of 0.26 nAm, suggesting ~10^4^ pyramidal neurons would need to be synchronously firing to create an observable strong PING rhythm (with an amplitude of 26 nAm) with the characteristic features described above.

### 3.2. Weak PING rhythms maintain characteristics of strong somatic inhibition and couple to bouts of high gamma frequency activity

While instructive for understanding how PING mechanisms are reflected in pyramidal cell dendritic CD signals, we considered the strong PING signals in Figure 2A to be unrealistic compared to experimentally observed CD recordings. In this section, we considered so-called “weak PING” rhythms where fewer E cells participated on each cycle of the rhythm with less coherence, which created signals that were less regular and likely more realistic. We show that weak PING rhythms produced a smaller amplitude, noisier signal but retained characteristic CD features reflective of backpropagating action potentials and strong somatic inhibition.

To simulate weak PING rhythms, instead of driving E cell somata with tonic depolarizing currents as in strong PING, stochastic Poisson-distributed synaptic inputs were used, causing E cells to spike randomly. The weak PING rhythm was initiated by a noisy barrage of E cell spiking that recruited coordinated I cell spiking, as shown in Figure 2B. Fewer E cells spikes were required to elicit spiking in a population of I cells by increasing the synaptic strength of E-I connections in the model (see Methods for details). As in strong PING, the sharp downward deflections of the CD waveform were caused by gamma periodic inhibition at the E cell somata, which suppressed most E cell spiking and positive CD deflections on each cycle of the oscillation. Only after the GABA_A_-ergic inhibition wore off did another sufficiently coherent barrage of excitatory activity emerge to resume the next cycle of the oscillation. GABA_A_-ergic synapses between I cells helped to coordinate I cell activity, which was not necessary for coherent I cell spiking in the strong PING model.

While coherent rhythmic activity is evident in the I cell spiking rasters of Figure 2B (top panel), the rhythm is now less obvious in the E cell spiking during weak PING, with the spike histogram showing a broader distribution of E cell spike timing on each cycle of the oscillation (Figure 2B, second panel), as compared with the strong PING case. Despite the weak E cell coherence, PING rhythms emerged in the current source signal from the E cells because of strong, coherent I-E somatic inhibition. The positive amplitude deflections in the weak PING current dipole still reflected currents generated by backpropagation of action potentials in the pyramidal cell dendrites, as in strong PING (Figure 2A), but they were temporally broader from the E cell spiking variability, which led to more sinusoidal oscillations. Here, excitatory cells in the weak PING model spiked with an average rate of 8.2 ± 3.3 Hz, in contrast to the strong PING simulations, in which excitatory cells spiked on each cycle of its 36 Hz oscillation. Despite the fact that individual E cells did not spike with gamma periodicity during weak PING, the net GABA_A_-ergic currents onto the E cell somata were prominent enough to create a rhythm in the somatic currents (Figure 2B, second panel) and in the corresponding net current dipole signal (Figure 2B, third panel).

Due to reduced participation and less coherent activity among the E cells in weak PING, the amplitude of the CD signal (maximal amplitude of 0.019 nAm) was 10 times smaller than in the strong PING case. This leads to the estimate that, in order to observe gamma frequency rhythms generated by these weak PING mechanisms in measured current dipole (~10 nAm), on the order of ~10^5^ pyramidal neurons would need to be gated coherently by somatic GABA_A_-mediated inhibition, assuming for simplicity that the fraction of participating cells increases linearly with the size of the network.

The GABA_A_-mediated PING rhythms described here created a specific characteristic feature in the CD signal that may help distinguish underlying PING mechanisms from alternative mechanisms that could also generate gamma frequency oscillations, such as those driven by thalamic inputs, which we report below. The characteristic feature in weak PING consisted of a broad positive deflection followed by a fast negative deflection that can be quantified by comparing the slope of the rising and falling phases of the oscillation. The CD waveform for the weak PING rhythm is shown in Figure 3, with maxima (red) and minima (green) marked for each cycle of the oscillation. For each cycle of the oscillation, we calculated a slope ratio (ϕ_*i*_), comparing the slope of the rising trough to peak vs. the slope of the falling peak to trough (see Methods). Over all cycles of the oscillation, we calculated the mean and standard error of the slope ratio (Φ). For weak PING oscillations, Φ was significantly less than 1 (0.61 ± 0.09, *p* < 0.05, one-sided *t*-test), which meant that the rising slope of the oscillation was slower than that of the decaying slope. This value reflected the decay time constant of the inhibition coupled to the fact that, for a given cycle of the gamma oscillation, the aggregation of E cell spikes built up over a relatively longer window of time before the fast, coordinated spiking activity of the inhibitory cell populations rapidly pulled current flow down the pyramidal neuron dendrites. We show below that this result is in contrast to subthreshold driven gamma rhythms, in which Φ was significantly greater than 1.

### 3.3. High gamma frequency oscillations emerged from characteristic waveforms within weak PING

**Figure 4:**
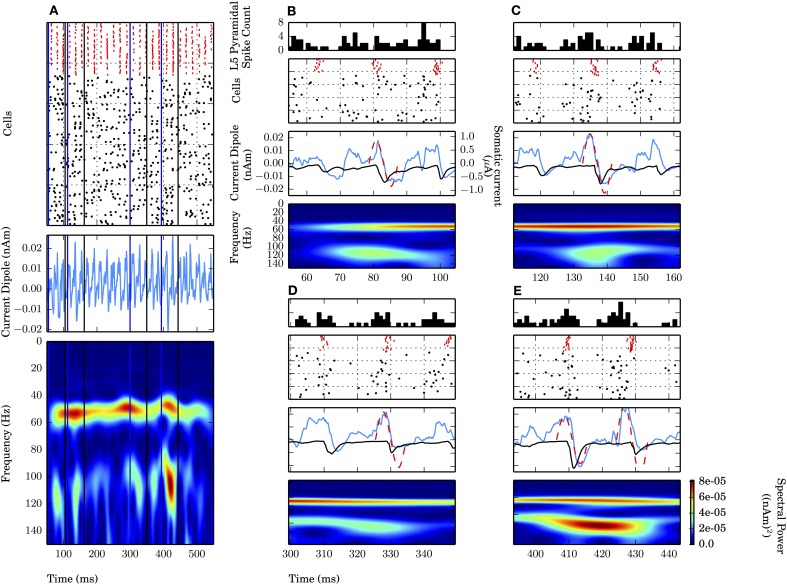
**High gamma frequency (100–120 Hz) oscillations due to specific waveform not separate oscillatory process or uncorrelated spiking.** Four epochs **(B–E)** of an example weak PING simulation (see text). Mean rate of E cell spikes in this example is 11.7 ± 2.8 Hz. PSTH Δt_*bin*_ = 1 ms. **(A)** Weak PING simulation, from top to bottom: spike rastergram of 100 E cells (black) and 35 I cells (red); current dipole (nAm); Morlet spectrogram of current dipole. Weak PING oscillation at 52 Hz (count 26 inhibition-mediated cycles in 500 ms in raster), Morlet spectral power (P_M_) of 7.69×10^−5^ (nAm)^2^ at 54 Hz in this band. Epochs of high gamma frequency activity denoted by lines for start times (blue) and end times (black) for 50 ms interval centered around max. P_M_. In each of **(B–E)**, from top to bottom: E cell spike PSTH; spike rastergram for E cells (black) and I cells (red); current dipole (nAm, blue) and schematic sinusoids depicting single cycles of high gamma frequency oscillations that emerged in the spectrograms (dotted red line), with somatic GABA_A_-mediated currents (μA, black). **(B)** Time interval of 54.5–104.5 ms. P_M_ = 4.34×10^−5^ (nAm)^2^ at *f* = 115 Hz at 79.6 ms. **(C)** 111.9–161.9 ms. P_M_ = 4.22×10^−5^ (nAm)^2^ at *f* = 114 Hz at 136.9 ms. **(D)** 299.4–349.4 ms. P_M_ = 3.79×10^−5^ (nAm)^2^ at *f* = 109 Hz at 324.4 ms. **(E)** 393.4–443.4 ms. P_M_ = 8.18×10^−5^ (nAm)^2^ at *f* = 106 Hz at 418.4 ms.

Notably absent during the weak PING simulations was the persistent, broadband high frequency spectral energy seen on all cycles of the strong PING oscillations. However, in simulations of weak PING, we observed high gamma frequency (~106–115 Hz) bouts that were mechanistically distinct from the artifactual broadband high frequency activity seen in strong PING (compare spectrograms in Figures 2A,B). The epochs were tied to 1–2 cycles of the weak PING oscillation, were not harmonics of the low gamma frequency signal, and did not reflect a separate gamma frequency process apart from the dominant lower frequency signal (Figure 4). Here, we discuss the mechanisms creating these high gamma frequency epochs.

Four of the high frequency epochs were examined (Figures 4B–E). The spiking rastergram, current dipole, and spectrogram for a simulation of weak PING is shown in Figure 4A, where the start of each epoch is marked with a blue line, and the end of the epoch is delineated in black. All of the high frequency epochs had a characteristic CD waveform consisting of a single (or double, as in Figure 4E) cycle of an oscillation with a period ranging from 8.7 to 9.4 ms (corresponding to frequencies from 106 to 115 Hz), marked schematically with a red dotted sinusoid in panels **B–E** (summarized in Table 2).

**Table 2:** **Epochs of high frequency activity**.

***t*_max_ (ms)**	***f* (Hz)**	**P_M_ [(nAm)^2^]**	**Subfigure**
79.6	115	4.34×10^−5^	B
136.9	114	4.22×10^−5^	C
324.4	109	3.79×10^−5^	D
418.4	106	8.18×10^−5^	E

This shape emerged from a consistent pattern of spiking activity in the underlying E-I network. Specifically, on each bout of high frequency activity, as the inhibition from the previous cycle wore off, an initial barrage of E cell spikes emerged, causing a brief, low amplitude positive deflection in the CD signal caused by the random E cells spikes. However, the excitation was not sufficient to elicit coordinated I cell spiking. As the prior cycle of inhibition continued to wear off, additional stochastic excitatory input eventually broke through the inhibition and elicited enough excitatory spikes to create a larger amplitude positive deflection in the CD signal and drive the next cycle of coordinated inhibition (spike rasters for the I (red) and E (black) cells are shown with a peristimulus time histogram (PSTH) of the E cells on top). In turn, the coordinated inhibition caused a sharp negative deflection (somatic GABA_A_-ergic currents are shown in black on top of blue CDs in Figures 4B–E middle panels). The temporal dynamics of the aggregation of excitation and subsequent inhibition led to the characteristic high gamma frequency waveform, depicted schematically with a red dashed sinusoidal curve overlaid onto CD waveforms and was reflected by increased high frequency spectral power consistent with the period of activity (Figures 4B–E). Most often, these high frequency oscillations occurred as a single cycle, but sometimes two cycles were formed, with an increased peak in spectral power directly between the cycles (Figure 4E).

Such short bouts of high gamma frequency activity were coupled to the ongoing weak PING oscillation and did not reflect a distinct PING oscillation separate from the lower gamma frequency rhythm. This high frequency activity was inconsistent with harmonic activity of the lower frequency ~54 Hz oscillation seen in Figure 4A, as the high frequency activity was temporally transient and varied inconsistently in both spectral power and peak frequency in each bout relative to the lower gamma oscillation. Spectral power due to uncoordinated E cell spiking was ruled out as the source of these epochs, as the frequency of those events was countably higher than the ~100 Hz spectral power, and the events were relatively low amplitude compared to the dominant oscillation.

### 3.4. Current dipole gamma oscillations are dominated by L5 activity

All of the prior simulations were performed simulating only L5. Here, we investigated the influence of a weak PING rhythm in L5 along with a simultaneous, independent weak PING rhythm in L2/3. Like the model of L5, the model of L2/3 also contained pyramidal neurons with apical dendrites whose longitudinal intracellular currents were used to calculate the equivalent current dipole (see Methods). We show that the activity in L5 dominates the net current dipole masking gamma in L2/3, due to the longer length of the apical dendrites in L5 pyramidal neurons.

**Figure 5:**
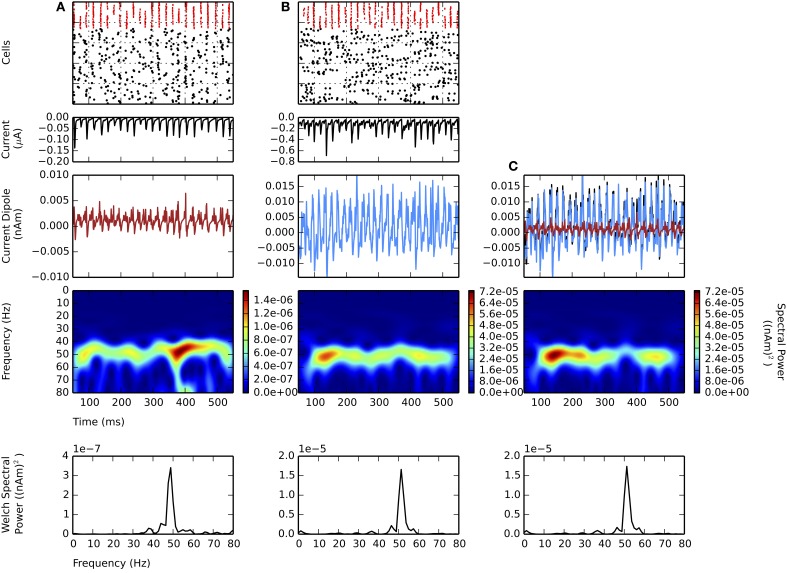
**Larger amplitude L5 weak PING rhythms dominate L2 weak PING rhythms in current dipole due to longer E cell dendrites in L5.** From top to bottom: spike rastergram of E cells (black) and I cells (red); aggregate GABA_A_-ergic current contribution to E cell somata (μA, black); net current dipole for E cells in layer (nAm); Morlet spectrogram of net current dipole [(nAm)^2^]; stationary Welch periodogram for net current dipole [(nAm)^2^]. g_*pois*_ = 0.75×10^−2^ mS/cm^2^. Simulations shown from 50 to 550 ms. **(A)** Independent weak PING in L2 shows ~50 Hz activity in Morlet spectrogram. Max. P_W_ of 3.4×10^−7^ (nAm)^2^ at 48.8 Hz. Max. value in net current dipole for 100 E cells in L2 was 6.0×10^−3^ nAm. **(B)** Independent weak PING in L5 also shows ~50 Hz activity in Morlet spectrogram. Max. P_W_ of 1.7×10^−5^ (nAm)^2^ at 51.2 Hz. Max. value in net current dipole for 100 E cells in L5 was 1.9×10^−2^ nAm. **(C)** Aggregate current dipole (black trace, mostly obscured by L5 current dipole) calculated as linear sum of net current dipoles from L2 (red) and L5 (blue). Morlet spectrogram shows ~50 Hz spectral power, but max. P_W_ of 1.7×10^−5^ at 51.2 Hz, which is same frequency as that in L5.

Figure 5 shows independent weak PING rhythms generated in L2/3 (panel **A**) and L5 (panel **B**), and the aggregate sum for the entire network (panel **C**). The mechanism generating the rhythm in L2/3 was analogous to that in L5, with the mean individual spiking rate for L2/3 pyramidal cells tuned so that they were not significantly different from the mean spiking rate in L5 (L2/3: 7.8 ± 1.4 Hz; L5: 8.2 ± 3.3 Hz; g_*pois*_ of 0.75×10^−2^ in L5), and the maximal frequencies in the CD spectrograms were both near 50 Hz.

The maximal amplitude of the CD in L2/3 (6.0×10^−3^ nAm) was 3 times smaller than L5 (19×10^−3^ nAm), mostly accounted for by the greater length of the L5 pyramidal cell dendrites. Thus, the net sum current dipole (shown in black in Figure 5C) was dominated by the L5 component (blue), which washed out the contribution from the lower amplitude L2/3 component (red). The dominance of L5 was further corroborated by separate simulations of random, uncoordinated excitatory spiking (non-gamma periodic) in L5 that also obscured ongoing weak PING in L2/3 (data not shown).

### 3.5. Subthreshold 30–100hz oscillations require coherent input and are mechanistically distinct from weak PING

While PING mechanisms are the most commonly cited origin of gamma frequency rhythms, particularly for the lower band, many other mechanisms could create gamma periodic activity, and an important question is whether there are distinguishing features of the signal that can help identify the underlying mechanisms. Here, we investigated an alternatively proposed mechanism to PING-type, I cell-mediated gamma rhythms in neocortex, namely gamma periodic inputs to neocortex from an exogenous network (e.g. thalamus) (Ribary et al., 1991; Llinás and Ribary, 1993; Castelo-Branco et al., 1998; Staudigl et al., 2012). Two specific types of exogenous drive could induce gamma in neocortex: a proximally-targeting drive from lemniscal thalamus and a distally-targeting drive from non-lemniscal thalamus (Jones, 2001). Here, we investigated the impact of subthreshold drive—in proximal only and combined proximal and distal projection patterns—on characteristic features of the CD signal. This investigation extends prior results suggesting roles of these projections in the generation of CD signals at lower frequencies than gamma (Jones et al., 2009). In each case, we found that subthreshold driven gamma rhythms required coherent thalamic inputs to create observable oscillations and had waveform features in the CD that were distinguishable from weak PING rhythms.

**Figure 6:**
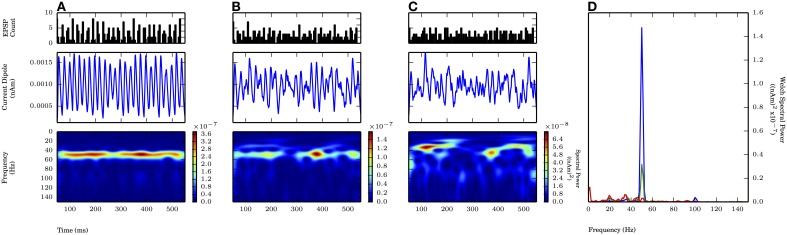
**Subthreshold oscillations required coherent inputs.** 50 Hz subthreshold oscillations generated by proximal AMPA-ergic EPSPs (see Methods). Each cycle of inputs comprised of 10 Gaussian distributed events with mean time corresponding to 20 ms oscillation period (50 Hz) and varying standard deviation (σ_*p*_). From top panel to bottom **(A–C)**: proximal input event PSTH; current dipole (nAm); Morlet spectrogram ((nAm)^2^ × 10^−7^). **(A)** σ_*p*_ = 2.5 ms: Persistent band of activity seen in Morlet spectrogram, max. P_W_ of 1.4×10^−7^ (nAm)^2^ at 50.1 Hz (blue in **D**). **(B)** σ_*p*_ = 5.0 ms: Spectral activity degraded compared to **(A)** at ~50 Hz (note different scales in spectrogram), max. P_W_ of 3.2×10^−8^ (nAm)^2^ at 50.1 Hz (green in **D**). **(C)** σ_*p*_ = 7.5 ms: Spectral activity further degraded from **(B)**, no identifiable peak in Welch spectral power at or around 50 Hz. **(D)** Welch periodogram of current dipole in **(A)** (shown in blue), **(B)** (green), and **(C)** (red) demonstrates reduction in P_W_ at 50 Hz as σ_*p*_ was increased.

We first investigated gamma periodic drive (50 Hz) of the L5 pyramidal cells in a proximal only drive pattern, representing simulated lemniscal thalamic drive (Figure 6). Each cycle of the input drive consisted of independent bouts of 10 Gaussian distributed excitatory postsynaptic currents (EPSCs) with the standard deviation of the drive (σ_*p*_) on each cycle varied between 2.5 and 7.5 ms (Figure 6, top panel shows PSTHs of the drive, see Methods for details). This input induced subthreshold current flow up the pyramidal neuron dendrites every ~20 ms that then decayed to baseline to create an oscillation in the CD signal with maximal spectral power at 50 Hz (Figure 6A, middle and bottom panels). As σ_*p*_ was increased from 2.5 to 5.0 ms, the peak frequency was maintained, but Welch spectral power was reduced 77%. When σ_*p*_ was further increased to 7.5 ms, the 50 Hz peak was no longer distinguishable from noisy lower frequency activity (Figures 6A–C, bottom panels and Figure 6D). The dendritic currents creating these subthreshold rhythms were distinct from the weak PING rhythms shown in Figure 2B. Here, the subthreshold proximal drive created weak backpropagating current flow up the pyramidal neuron dendrites initiated by the synaptic currents that was smaller than currents created by backpropagation of action potentials caused by E cell spikes in weak PING rhythms. The amplitude of the subthreshold rhythms was an order of magnitude smaller than that of the weak PING rhythm, suggesting 10 times as many neurons would need to be driven in the subthreshold regime to create oscillations on the same order as weak PING. Specifically, these simulations predict that on the order of ~10^6^ neurons would need to be synchronously driven to create an observable signal on the order of 10 nAm.

With proximal only inputs, the downward deflections of the subthreshold rhythms reflected relaxation of the upward current flow rather than the strong pull of inhibitory somatic currents as in weak PING, and as a consequence, the downward deflections were temporally broader. This feature was quantified with slope ratio (Φ), as described for weak PING above, with the results summarized in Figure 9. Φ was calculated for values of σ_*p*_ at 2.5 and 5.0 ms (1.47 ± 0.07 and 1.57 ± 0.24, respectively) and were significantly greater than 1 in each case (for each: *p* < 0.05, one-sided *t*-test), demonstrating that the rising phases of the oscillation were faster than the subsequent decaying phases in the CD.

**Figure 7:**
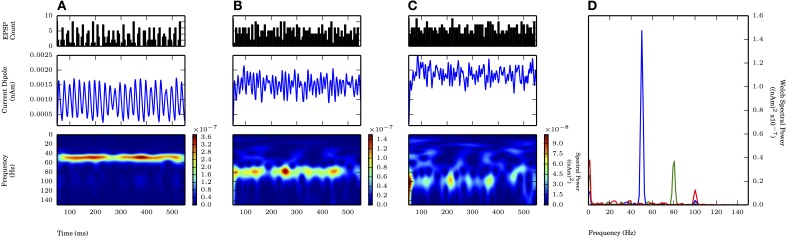
**Faster subthreshold oscillations break down at fixed standard deviation.** Subthreshold oscillations generated similarly as in Figure 6. Fixed σ_*p*_ of 2.5 ms. As frequency of input (*f*_prox_) increased, the spectral power decreased. **(A)** *f*_prox_ = 50 Hz: Same as Figure 6A. Max. P_W_ of 1.4×10^−7^ (nAm)^2^ (blue in **D**). **(B)** *f*_prox_ = 80 Hz: Less consistent band of activity at 80 Hz, compared to 50 Hz **(A)**. Max. P_W_ of 3.7×10^−8^ (nAm)^2^ at 80.6 Hz (green in D). **(C)** *f*_prox_ = 100 Hz: Less consistent band of activity at 100 Hz, compared to 50 Hz **(A)** and 80 Hz **(B)**. P_W_ of 1.3×10^−8^ (nAm)^2^ at 100.1 Hz (red in D). **(D)** Welch periodogram of current dipole in **(A)** (shown in blue), **(B)** (green), and **(C)** (red) demonstrates reduction in P_W_ as *f*_prox_ was increased.

We also tested whether subthreshold driven rhythms could be sustained at higher frequencies of drive, possibly representing an alternative mechanism to the high frequency (~100 Hz) oscillations seen in Figure 4. Figure 7 shows panels analogous to Figure 6, with the frequency of the periodic drive varied between 50, 80, and 100 Hz for a fixed σ_*p*_ of 2.5 ms. As the frequency increased, the input drive became less coherent (see PSTHs in the top panel of Figure 7), and the amplitude of the oscillations decreased. Φ for the 80 Hz rhythm was also significantly greater than 1 (1.32 ± 0.09, *p* < 0.05 one-sided *t*-test, see raw CD in Figure 7, for comparison see Figure 9). However, when the input frequency was 100 Hz, the spectral power decreased to the extent that the peak was no longer distinguishable from lower frequency noise (Figure 6D).

In total, the results of the proximal only drive suggest that for these subthreshold cortical rhythms to be observed, the driving input must be highly synchronous (less than 7.5 ms variance on each cycle of the input) and coherent across large volumes of cortex. Specifically, our simulations predict that on the order of ~10^6^ neurons need to be synchronously driven to create an observable 10 nAm signal.

### 3.6. Proximally driven subthreshold gamma enhanced by coherent distal drive

Next, we simulated 50 Hz gamma periodic subthreshold drive from simultaneous proximal and distal inputs targeting the L5 pyramidal cells. The results showed that ongoing proximally-driven gamma rhythm can be enhanced by a gamma periodic distal input, and the ongoing gamma was more robust to changes in the variance of the distal input on each cycle than to changes in input variance when only the proximal drive was present.

**Figure 8:**
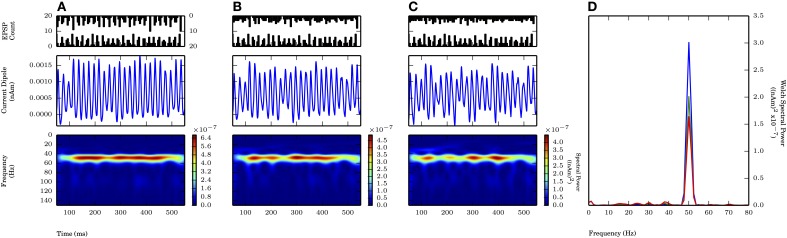
**Proximally driven subthreshold gamma enhanced with distal gamma periodic input and is robust to distal input noise.** Ongoing 50 Hz proximal input with σ_*p*_ fixed at 2.5 ms provided rhythmic 50 Hz activity in current dipole. Distal input of 50 Hz (offset 5 ms on each cycle from proximal input, see Methods) with varied σ_*d*_ at values of 2.5, 5.0, and 7.5 ms. Panel configuration same as Figures 6, 7. Max. P_W_ values all occurred here at 50.1 Hz. **(A)** σ_*d*_ = 2.5 ms: Persistent band of activity seen in Morlet spectrogram, max. P_W_ of 3×10^−7^ (nAm)^2^ (blue trace in **D**). P_W_ increased compared to proximal only inputs with σ_*p*_ of 2.5 ms in Figure 6A (1.4×10^−7^ (nAm)^2^). **(B)** σ_*d*_ = 5.0 ms: Band of activity persistent in Morlet spectrogram, max. P_W_ decreased to 2×10^−7^ (nAm)^2^ (green trace in **D**), as compared to **(A)**. Less severe drop in P_W_ occurred in change to σ_*d*_ with ongoing proximally driven gamma frequency oscillation (compare to Figure 6). **(C)** σ_*d*_ = 7.5 ms: Again, persistent band of activity in Morlet spectrogram. Max. P_W_ decreased to 1.6×10^−7^ (nAm)^2^ (red trace in **D**), but peak still distinguishable, provided by ongoing proximally driven gamma. **(D)** Welch periodogram of current dipole in **(A)** (shown in blue), **(B)** (green), and **(C)** (red) demonstrates small reduction in P_W_ as σ_*d*_ was increased, during an ongoing oscillation driven also by the proximal input. Spectral power in each simulation was evoked by proximally driven gamma, and reduction in P_W_ with increased σ_*d*_ was less severe than effect of changing σ_*p*_ in Figure 6.

On each cycle of the oscillation, both proximal and distal inputs consisted of independent rhythmic bouts of 10 Gaussian distributed excitatory post synaptic currents with the value of σ_*p*_ at 2.5 ms (motivated by the proximal drive only results, see Figure 6A) and values of the standard deviation of inputs on each cycle of the distal drive (σ_*d*_) of 2.5, 5.0, and 7.5 ms (Figure 8 PSTHs in top panels). The mean times for the distal input on each cycle followed that of the proximal input by 5 ms. This combination of 50 Hz proximal and 50 Hz distal inputs created a 50 Hz oscillation in the CD signal generated by current flow being driven up and subsequently down the pyramidal neuron dendrites via the proximal and distal synaptic drives, respectively (Figure 8A). Compared to proximal only simulations with the same σ_*p*_ of 2.5 ms, the spectral power was approximately twice as large here (cf. amplitude of blue traces in Figure 8D with Figure 6D). Given that these cortical network oscillations remained in the subthreshold regime, the amplitude of the oscillations was comparable to the proximal only case and still an order of magnitude smaller than in weak PING. As the distribution of the distal inputs on each cycle became broader (σ_*d*_ = 5.0, 7.5 ms), the maximal spectral power was reduced (Figures 8B,C). The reduction in power reflected the fact that the oscillations became noisier and less sinusoidal, despite being similar in amplitude to oscillations driven by inputs with σ_*d*_ of 2.5 ms and despite being dominated by the 50 Hz periodicity of the proximal drive. Here, the reduction in spectral power by changing σ_*d*_ from 2.5 to 5.0 ms was approximately 33% Figures 8A,B, shown in **D**). This power reduction was less than that seen for proximal only inputs where σ_*p*_ was changed from 2.5 to 5.0 ms (Figures 6A,B, shown in **D**), which suggested that the influence of broader distal input was less disruptive with an ongoing, proximal input-driven gamma rhythm present.

**Figure 9:**
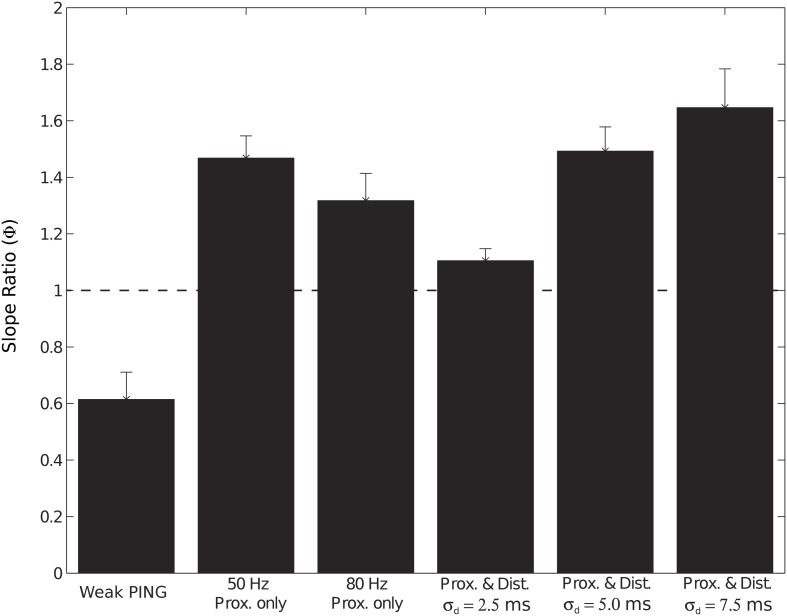
**Slope ratio (Φ) significantly less than 1 for current dipole from weak PING but significantly greater than 1 for subthreshold (sub.) simulations.** Φ was calculated as mean of the ratio (ϕ_*i*_) of rising slope to falling slope for each cycle *i* of CD rhythm (see Methods and Figure 3). Φ of 1 signifies that rising slope was equivalent to falling slope. Error bars represent standard error (s.e.) about the mean. Φ values (relevant CD waveform in parentheses) found in: weak PING, 0.61 ± 0.09 (Figure 3); sub. 50 Hz proximal only, 1.47 ± 0.07 (Figure 7A); sub. 80 Hz proximal only, 1.32 ± 0.09 (Figure 7B); sub. 50 Hz proximal and distal, σ_*d*_ = 2.5 ms, 1.1 ± 0.04 (Figure 8A); sub. 50 Hz proximal and distal, σ_*d*_ = 5.0 ms, 1.5 ± 0.04 (Figure 8B); sub. 50 Hz proximal and distal, σ_*d*_ = 7.5 ms, 1.65 ± 0.14 (Figure 8C).

We investigated the impact of the additional distal drive on Φ. The addition of distal drive caused a sharper downward deflection of the subthreshold driven oscillation, in comparison to oscillations driven with only proximal inputs. However, with σ_*d*_ values of 2.5, 5.0, and 7.5 ms, Φ was still significantly greater than 1 (1.1 ± 0.04, 1.5 ± 0.04, and 1.65 ± 0.14, respectively; *p* < 0.05 one-sided *t*-test), in contrast to PING rhythms in which strong somatic inhibitory currents resulted in Φ significantly less than 1. These results for Φ are summarized in Figure 9.

In total, the results of the subthreshold thalamic drive simulations suggested that, for these rhythms to be observed, the driving input must be highly synchronous in time (σ_*p*_ and σ_*d*_ must be less than 7.5 ms on each Gaussian distributed cycle of the input) and coherent across large volumes of cortex. For an equivalently sized network, the amplitude of the oscillations created by subthreshold oscillations were ~10 times smaller than the spiking weak PING rhythms. Further, our results predicted that the mechanism of observed gamma rhythms in CD signals may be distinguished by the slope ratio (Φ) of the oscillations: if Φ is significantly less than 1, PING mechanisms may be responsible, and when Φ is significantly greater than 1, these results suggest that the rhythm can be generated by subthreshold thalamic drive.

## 4. Discussion

In this paper, we used biophysically principled computational modeling of current dipoles in neocortex to explore for the first time how mechanisms of gamma frequency rhythms can be expressed in these macroscopic signals that underlie human MEG/EEG recordings and estimated from ECoG data (Malmivuo and Plonsey, 1995). In the spirit of Siegel and König (2003), our goal was to move beyond the current classification of this rhythm in humans, which relies on a frequency definition over a broad frequency range (30–150 Hz), to a mechanistic description. Our results show that alternative mechanisms of gamma generation create distinguishable features in the current dipole waveform that can help to disambiguate the underlying cellular level generators.

Specifically, we found that gamma frequency rhythms created by well-established weak PING mechanisms created a current dipole waveform that was characterized by sharp downward deflections, due to strong inhibition at the soma. In contrast, gamma periodic rhythms created by driven subthreshold excitatory synaptic input from upstream sources created a waveform with a broader rise and fall (Figure 9). Further, our results predicted that PING mechanisms create larger amplitude signals than subthreshold driven rhythms, due to strong backpropagation of action potentials in the dendritic currents of pyramidal neurons that contribute to the current dipole signals.

Additionally, we showed that the activity of L5 dominates the current dipole signal and can mask the presence of gamma in L2/3 (Figure 5). Lastly, our results led to a novel prediction on the origin of high gamma frequency oscillations (100–150 Hz) in current dipole signals. We showed that single cycles of high gamma periodic oscillations emerged in the current dipole waveform during some cycles of weak PING oscillations, and these were created by a narrow (~5 ms), random barrage of backpropagating action potentials that initiated the rise of the high frequency waveform that was followed by the somatic inhibition to produce the negative deflection of the oscillation (Figure 4C). These bouts of high gamma frequency activity did not reflect a separate, ongoing high frequency oscillation, were not due to uncorrelated spiking, and were also unique from subthreshold oscillations. Specific frequencies were fixed in some simulations here to compare subthreshold and spiking oscillations, but in general these networks can exhibit a range of gamma frequencies from PING mechanisms that are dependent upon the synaptic parameters and strength of input drive. Though not shown explicitly here, we would expect that the results derived from the spiking PING simulations here would be generalizable to networks operating at other gamma frequencies.

### 4.1. Prior models of gamma oscillations have not simulated current dipole

Many prior modeling studies have investigated features of gamma rhythms in neocortical networks produced in spiking or simulated LFP signals (Traub et al., 1997; Börgers et al., 2005; Lee et al., 2009; Vierling-Claassen et al., 2010), as well as in mean field models (David and Friston, 2003; Deco et al., 2008). To our knowledge, no prior model of gamma rhythms has ever explicitly simulated the cellular level biophysics of macroscopic current dipole signals, such as those recorded by MEG or EEG. PING mechanisms, as described in the methods and results, have been established in many simplified biophysical models that used single compartments to represent both excitatory and inhibitory cells (Börgers et al., 2005; Lee et al., 2009). These minimal models were sufficient to demonstrate that the time constants of GABA_A_-mediated currents can create gamma frequency rhythms. Other models of gamma rhythms have reached similar conclusions using multi-compartment models of pyramidal neurons with realistic geometries (Traub et al., 2005; Vierling-Claassen et al., 2010) but have not explicitly estimated current dipole.

In our results, PING rhythms were also paced by GABA_A_-mediated currents, consistent with prior work. By explicitly simulating current dipole signals, we found that dendritic current flow can maintain PING-mediated gamma rhythms and enable direct comparisons to macroscopic current dipole data from humans. This approach also helped to suggest a novel prediction on the origin of high frequency gamma epochs that were coupled to the lower frequency PING rhythm (Figure 4), a feature that would not have been captured without considering current dipole. Further, our simulations allowed us to study gamma rhythms generated by coordinated subthreshold exogenous drive to distinct neocortical layers.

### 4.2. Importance of considering unfiltered time courses in interpreting gamma mechanisms

Our results suggest that inferences on the underlying cellular mechanisms of gamma rhythms in human macroscopic signals can be made by considering features of raw unfiltered signals. Typically, human imaging data of gamma rhythms is shown after a frequency filter (e.g. Fourier transform or wavelet transform) has been performed. Many studies that present time series data first apply a band pass filter in a gamma band of interest and often over a broad range. Given that low and high gamma oscillations can emerge from distinct cellular level mechanisms, our result suggest that, when interpreting the generator of gamma and its impact in information processing, the frequencies should not be considered as a single band, and raw signal time series should be considered in conjunction with frequency analysis and population measures (Lin et al., 2004). Furthermore, investigation of the unfiltered waveforms can differentiate gamma frequency activities that emerge as spurious artifacts created by applying frequency filters to non-oscillatory signals (Kramer et al., 2008; Buzsáki and Wang, 2012) or as single cycle oscillations embedded in other rhythms, as shown for high gamma in Figure 4. Several studies report gamma activity in current source signals (Brookes et al., 2004; Hoogenboom et al., 2006; Roux et al., 2012). However, we cannot speculate on how our predictions apply to that data since interpretation of the mechanisms identified based on our results would require raw, unfiltered data.

Whether or not similar features in gamma rhythms as those described here emerge in MEG/EEG sensor space or surface electrodes before the inverse localization is applied is an important and open question beyond the scope of the current study. We conjecture that the strong negative deflection features present in PING rhythms will persist at the sensor level, particularly in MEG recordings, which are not distorted by cerebrospinal fluid and the skull and are less susceptible to source orientation cancellation effects (Hämäläinen et al., 1993).

### 4.3. Laminar interactions of gamma frequency rhythms

Our results showed gamma frequency activity in L5 networks dominate net current dipole signals, obscuring simultaneous gamma (or other activity) in a L2/3 network of the same size, due to the longer length of L5 pyramidal neuron dendrites (Figure 5). These results allow us to further predict that L2/3 gamma rhythms would only be observed in current dipole signals if the size of the network participating in the rhythms is significantly massive (see Estimates, below). Differentiation of separable contribution of L5 and L2/3 is not possible with current dipole source data alone and would require simultaneous laminar recordings.

There are multiple possible modes of gamma related to stimulus processing that may have laminar specificity. Stimulus evoked gamma activity has been shown to have laminae-specific corticocortical interaction (Roberts et al., 2013), while gamma related to attention has been found to be specific to superficial layers in the ventral visual system (Buffalo et al., 2011). Attention-related changes in gamma rhythmicity have been suggested to be mediated by cholinergic modulation (Börgers et al., 2005; Deco and Thiele, 2011), but separate studies using MEG have observed that gamma oscillations were not increased during cholinergic activation associated with attention (Bauer et al., 2012). In the latter study, Bauer et al. suggested that the lack of cholinergic modulation of gamma power may be related to differential laminar effects of cholinergic action. Our results may provide support for this view: if cholinergic modulation affected gamma oscillations that are primarily localized to superficial layers, our model results suggest that detection of that activity by MEG may be more difficult when layer 5 is engaged at a sufficient level. Another study using MEG has observed gamma band increases due to spatial attention, in which the authors suggested that their experimental paradigm was well suited to observe gamma-related attentional changes specifically (Koelewijn et al., 2013). Our results may provide a number of possible explanations for why gamma was observable in this study: strong engagement of superficial gamma oscillations due to spatial attention may have created a superficial network that was sufficiently large to be detected by MEG. An alternative hypothesis that is consistent with our results would suggest that gamma band increases related to attention may have increased in deep layers, either directly or via feedforward drive of deep gamma oscillations from superficial laminar gamma generators.

A differentiation has been made in the role of evoked (i.e., stimulus-locked) vs. induced (not stimulus-locked) gamma activity. While evoked gamma is by definition created by the stimulus and drives granular and infragranular layers, induced gamma has been suggested to be mediated by top down mechanisms (Tallon-Baudry and Bertrand, 1999; Tallon-Baudry, 2009), which are also related to modulatory inputs to superficial cortex (Weinberger, 1998; Weinberger et al., 2013). Tallon-Baudry and colleagues have suggested that early evoked gamma rhythms in human EEG/MEG are created by current dipoles generated by deep pyramidal neurons with long dendrites, whereas induced gamma rhythms might be created by more superficial and/or radially oriented sources (perpendicular to the skull, which are not detected by MEG) (Tallon-Baudry and Bertrand, 1999; Tallon-Baudry et al., 1999). These conclusions are based on their observations that evoked gamma can be recorded at the sensor level with both EEG and MEG, while induced gamma is not recorded with MEG (Tallon-Baudry et al., 1997; Uhlhaas et al., 2011). While the observation that gamma is particularly difficult to detect by MEG can be overcome by choosing optimized stimulus paradigms (Hoogenboom et al., 2006), Tallon-Baudry et al. have also proposed a specific radial source model as a possibility for the origin of their induced gamma observations (Tallon-Baudry et al., 1999; Tallon-Baudry and Bertrand, 1999). Our work extends this prediction suggesting a tangentially oriented L2/3 gamma could be present but masked in the net current dipole signal that is dominated by L5. The radial source model (Tallon-Baudry et al., 1999) was also motivated by the fact that gamma rhythms have been recorded without polarity reversal in laminar LFP recordings from anesthetized and behaving cats (Steriade and Amzica, 1996; Steriade et al., 1996a,b), suggesting they may not be created by a single large oscillatory dipole. However, there may be micro sink and source pairs across different layers (Tallon-Baudry et al., 1997), and in particular in L2/3, as has been reported in *in vivo* current source density signals from awake macaques (Lakatos et al., 2005).

Laminar recordings from cortical slices have shown that, under certain pharmacological manipulations, synchronous gamma can emerge across all layers, but multiple gamma rhythms can also exist with different frequencies in distinct layers and is dependent upon interlaminar interactions (Lakatos et al., 2005; Oke et al., 2010; Ainsworth et al., 2011, 2012). Reciprocal influence between L2/3 and L5 (Thomson and Bannister, 2003) could also impact the expressed gamma rhythms in CD signals. Our results suggest that functionally relevant activity in superficial layers may not be detectable in current source signals. Here, our results did not account for interlaminar connections, since our goal was to investigate their differential contribution, but we acknowledge that further work considering interlaminar interactions may be critical in connecting the mechanisms of gamma to their role in information processing.

### 4.4. Precision of subthreshold inputs

In this paper, we simulated subthreshold driven inputs to the cortical networks in proximal or combined proximal and distal input patterns as an alternative mechanism to PING rhythms. A motivation for this investigation was evidence for the emergence of cortical gamma rhythms related to gamma periodic activity in the thalamus (Ribary et al., 1991; Llinás and Ribary, 1993; Castelo-Branco et al., 1998; Staudigl et al., 2012), which could contact neocortex at superficial or granular sites (Jones, 2001). Further, macroscopic imaging signals can be produced by the subthreshold synchronous activity of large populations of pyramidal neurons, in contrast to large populations of synchronously spiking neurons (Jones et al., 2009; Zhu et al., 2009).

When exogenous drive was applied to the cortical network in our simulations, the ability of the subthreshold cortical oscillations to follow the drive demonstrated a trade-off between the frequency and the variance of the drive. This effect was demonstrated separately for frequency (Figure 7) and standard deviation (Figure 6). Our results suggested that with proximal only drive, the emergence of gamma required tight coherence in the input on each cycle, such that gamma power was lost when the standard deviation of the drive was as large as 7.5 ms. Combined proximal and distal drive induced a gamma rhythm with greater spectral power but also required coherent input on each cycle. With coherent proximal drive (σ_*p*_ = 2.5 ms), gamma rhythms persisted for larger variation in the distal coherence (σ_*d*_ = 5.0, 7.5 ms), suggesting that thalamocortically driven gamma to proximal dendrites may persist in the CD signal even with less coordinated distal inputs.

While the proximal drive in our model is most similar to lemniscal thalamocortical input, the distal drive could come from non-lemniscal thalamus or intracortical sources, either of which could oscillate at gamma. Activity at gamma frequencies has been observed in many thalamic areas, such as lateral geniculate, centrolateral, and posterior nuclei, simultaneously with gamma in cortical EEG signals (Ghose and Freeman, 1992; Canu et al., 1994; Steriade and Amzica, 1996; Steriade et al., 1996b). The data in these papers suggests a specific phase relationship between thalamic spiking and cortical gamma on each cycle of the oscillation and a temporal distribution of the thalamic spiking on the order of 5 ms on each cycle, consistent with our chosen model parameters.

Recent studies of 60–100 Hz gamma oscillations in motor cortex have shown coherence with subcortical basal ganglia structures, with evidence that gamma in basal ganglia may drive cortical gamma, suggesting interactions through the thalamus (Litvak et al., 2012; Cheyne and Ferrari, 2013; Jenkinson et al., 2013). In these studies of the motor system, gamma activity is observed in post-movement periods and is promoted by dopaminergic therapy in Parkinson's Disease patients with corresponding improvements in motor function (Jenkinson et al., 2013). It has also been proposed that the long-range gamma coherence from basal ganglia to cortex is related to arousal and motor vigor (Litvak et al., 2012), but the mechanisms promoting this coherence are unknown. Our results suggest possible means to help disambiguate cortical gamma driven by basal ganglia-thalamocortical drive from local PING mechanisms possibly coordinated across structures by common neuromodulatory influences, such as acetylcholine, which has been shown to promote transitions to GABA-mediated gamma rhythms (Pinto et al., 2003).

### 4.5. Estimates on the spatial scale of recordable gamma rhythms

Historically, gamma rhythms are known to be more difficult to record in human macroscopic MEG/EEG signals than lower frequency rhythms (Dalal et al., 2009). However, under certain task conditions and analysis methods, gamma rhythms have been repeatably recorded at the sensor (Tallon-Baudry et al., 1997; Hoogenboom et al., 2006) and source localized level (Brookes et al., 2004; Freunberger et al., 2007; Brookes et al., 2008) and most commonly in post-stimulus evoked and induced responses (Tallon-Baudry et al., 1997). The amplitude of these rhythms is orders of magnitude smaller than lower frequency rhythms, likely due to the restricted spatial scale over which they emerge (Steriade et al., 1996b; von Stein and Sarnthein, 2000; Rols et al., 2001) and cancellation effects that contribute to decreased magnitudes in macroscopic imaging signals (Hämäläinen et al., 1993).

The biophysically principled design of the model utilized here allowed us to make predictions on the size of the pyramidal network that would need to be simultaneously activated to create source localized primary current dipole gamma rhythms of the specific amplitude in appropriate, observable units (e.g. nAm). Reported magnitudes of gamma oscillations in current source signals range from 1 to 30 nAm (Freunberger et al., 2007; Brookes et al., 2011), and in general for MEG, the minimum for observable stimulus-evoked source signals is on the order of 10 nAm (Hämäläinen et al., 1993; Murakami and Okada, 2006; Jones et al., 2007; Nevalainen et al., 2008; Jones et al., 2009, 2010). Our results predicted gamma rhythms of ~19 nAm produced by weak PING mechanisms required participation of a network of ~10^5^ L5 pyramidal neurons with sparse spiking, while subthreshold driven rhythms required a network an order of magnitude larger to create comparable signals: ~10^6^ pyramidal neurons for 10 nAm. A network in L2/3 producing similar amplitude gamma signals would have to be approximately 3 times as large as that in L5. The number of neurons in 1 mm^2^ cortical area has been estimated to be ~10^5^–10^6^ (Rockel et al., 1980; Herculano-Houzel et al., 2008; Carlo and Stevens, 2013), which may be variable across mammalian species (Haug, 1987; Herculano-Houzel et al., 2008) but of which 75–80% of which are pyramidal neurons (Johansson and Lansner, 2007). Thus, weak PING mechanisms could coordinate an observable signal across approximately 0.8 mm of neocortex, roughly the size of a cortical column (0.5 mm), while subthreshold rhythms require approximately 8 mm of synchronous activity, roughly half the estimated size of the human D3 finger map [~16 mm, Sanchez-Panchuelo et al., 2010]. These estimates are consistent with reports of spatial coherence of gamma activity from subdural grids in visual cortex of monkeys (Rols et al., 2001) and somatosensory cortex of humans (Meador et al., 2002).

### 4.6. Coupling between high gamma and lower frequency activity

Numerous studies have reported that neocortical gamma rhythms across a range of frequencies are coupled to the phase of lower frequency rhythms, most commonly theta (4–8 Hz) (Lakatos et al., 2005; Canolty et al., 2006; Doesburg et al., 2012; van der Meij et al., 2012) or alpha (8–12 Hz) (Palva et al., 2005; Jensen and Mazaheri, 2010; Voytek et al., 2010; Maris et al., 2011). Our results give a novel interpretation of the origin of high gamma activity by showing how single cycles of high gamma can emerge embedded in a slower gamma frequency rhythm but were not a separate rhythmic process or representative of uncorrelated spiking activity. Rather, the high frequency activity emerged from the envelope of the current dipole signal waveform created by a bout of ~5 ms excitatory spiking followed by ~5 ms somatic inhibition that created a single ~100 Hz positive/negative oscillation (see Figure 4). In this study, these high gamma bouts were tied to a lower frequency gamma rhythm induced by local weak PING mechanisms, without external rhythmic drive. However, we conjecture that analogous bouts of ~5 ms excitation followed by ~5 ms inhibition could be modulated by periodic exogenous excitatory input at a slower frequency such as alpha or theta. For example, if the local network received excitatory synaptic drive at an alpha frequency of 10 Hz (i.e. every 100 ms), it is possible that this input could integrate to create brief bouts of excitatory spiking followed by inhibition, inducing a high frequency oscillation similar to that shown in Figure 4. This mechanism would create brief periods (~5 ms) of highly synchronous excitation occurring at specific phases of the alpha oscillation.

Prior theories suggest gamma oscillations coupled to theta or alpha oscillations involve amplitude modulation of ongoing gamma rhythms at a broad range of frequencies (as long as the modulated frequency is higher than the carrier frequency) and that this is due to periods of increased excitability at peaks of the carrier cycle (Fries, 2005; Jensen and Colgin, 2007; Lakatos et al., 2008). Our prediction specifically suggests there are very brief bouts (~5 ms) of highly synchronous excitation within the slower oscillation that would be observed in current source signals as a high gamma oscillation. However, the proposed mechanism does not negate the possibility that longer periods around the peak of the lower frequency cycle could contain smaller sub-assemblies of heightened excitability, exhibiting a broad range local gamma rhythms that are not visible in the current source signal, which would be consistent with the prior theories based on invasive recordings.

In prior MEG and modeling work, we have shown that low frequency alpha and beta rhythms in current source signals can be reproduced by driven subthreshold pyramidal neuron current flow induced by interacting 10 Hz proximal and distal drive (Jones et al., 2009; Ziegler et al., 2010). In those studies, 6 × 10^6^ synchronous pyramidal neurons created subthreshold oscillations that were on the order of 100 nAm consistent with recorded low frequency MEG measured somatosensory rhythms that contained a complex of alpha and beta referred to as a mu-rhythm (Jones et al., 2009). An important future extension of this work will be to use the model to investigate the interaction between large-scale subthreshold rhythms and smaller sub-assemblies of spiking neurons that may oscillate via weak PING mechanisms or be driven to create single cycles of high gamma, as described above. However, the current spatial scale of our model does not allow for simulations of simultaneous spiking and subthreshold rhythms, as spiking in any pyramidal neuron will recruit spiking in the majority of neighboring neurons across the entire simulated grid. Thus, future model expansion will be necessary to investigate large and small scale interactions.

### 4.7. Conclusions

The results from our model simulations suggested that different mechanisms can produce oscillations in the gamma frequency band, and the nominal frequency alone was not sufficient to describe the activity. Based on our model results, we propose that identification of unique features of the temporal dynamics of the current dipole waveform can provide evidence to help disambiguate the underlying mechanisms. Further, differential expression of laminar gamma rhythms that may be important in cortical function may be difficult to assess from current source signals. Overall, understanding the mechanisms that generate these gamma frequency rhythms may be critical for a more thorough understanding of their function in human health and disease.

### Conflict of interest statement

The authors declare that the research was conducted in the absence of any commercial or financial relationships that could be construed as a potential conflict of interest.
